# Recognition of Emotions Using Multichannel EEG Data and DBN-GC-Based Ensemble Deep Learning Framework

**DOI:** 10.1155/2018/9750904

**Published:** 2018-12-13

**Authors:** Hao Chao, Huilai Zhi, Liang Dong, Yongli Liu

**Affiliations:** School of Computer Science and Technology, Henan Polytechnic University, Jiaozuo, China

## Abstract

Fusing multichannel neurophysiological signals to recognize human emotion states becomes increasingly attractive. The conventional methods ignore the complementarity between time domain characteristics, frequency domain characteristics, and time-frequency characteristics of electroencephalogram (EEG) signals and cannot fully capture the correlation information between different channels. In this paper, an integrated deep learning framework based on improved deep belief networks with glia chains (DBN-GCs) is proposed. In the framework, the member DBN-GCs are employed for extracting intermediate representations of EEG raw features from multiple domains separately, as well as mining interchannel correlation information by glia chains. Then, the higher level features describing time domain characteristics, frequency domain characteristics, and time-frequency characteristics are fused by a discriminative restricted Boltzmann machine (RBM) to implement emotion recognition task. Experiments conducted on the DEAP benchmarking dataset achieve averaged accuracy of 75.92% and 76.83% for arousal and valence states classification, respectively. The results show that the proposed framework outperforms most of the above deep classifiers. Thus, potential of the proposed framework is demonstrated.

## 1. Introduction

Emotion plays an important role in the daily life of human beings. Especially, peoples communicate more easily through emotional expression, and different emotional states can affect people's learning, memory, and decision-making. Therefore, recognition of different emotional states has wide application prospects in the fields of distance education, medicine, intelligent system, and human-computer interaction. Emotion recognition has recently been highly valued by researchers and has been one of the most important issues [1].

Emotional recognition can be performed by external features such as facial expressions and voice intonation [2–6]. It can also be performed according to changes of physiological signals such as electroencephalogram. Compared with physiological signals, facial/vocal expressions are easily affected by the external environment and the parameters easily vary in different situations. However, the emotion recognition results from EEG signals are relatively objective due to the fact that physiological signals are hard to be camouflaged. Therefore, the studies of associations between EEG activity and emotions have received much attention [7–9].

Emotion recognition is essentially a pattern recognition task, and one of the key steps is extracting the emotion-related features from the multichannel EEG signals. Various EEG features in time domain, frequency domain, and time-frequency domain have been proposed in the past. Time-domain features from EEG can identify characteristics of time series that vary between different emotional states. The statistical parameters of EEG series, such as mean, standard deviation, and power, were usually employed [10–12]. Frantzidis et al. used amplitude and latency of event-correlated potentials (ERPs) as features for emotion recognition [13]. In addition, Hjorth features [14, 15], nonstationary index [16], and higher order crossing features [17, 18] have also been utilized. Power features from different frequency bands from EEG signals are most popular in frequency domain techniques. The EEG power spectral density (PSD) in alpha (8–13 Hz) band was reported to be significantly correlated with the level of valence [19]. The EEG PSDs in delta (1–4 Hz) and theta (4–7 Hz) extracted from three central channels also contain salient information related to both arousal and valence levels [13]. Based on time-frequency analysis, the Hilbert–Huang spectrum (HHS) [20] and discrete wavelet transform method [21, 22] were proposed in the emotion classification tasks. The above research shows that time domain characteristics, frequency domain characteristics, and time-frequency characteristics of EEG signals can provide salient information related to emotional states separately.

Usually, machine learning methods are used to establish emotion recognition models. Samara et al. fused statistical measurements, band power from the *β*, *δ*, and *θ* waves, and high-order crossing of the EEG signal by employing a support vector machine (SVM) as the classifier [23]. Jadhav et al. proposed a novel technique for EEG-based emotion recognition using gray-level co-occurrence matrix- (GLCM-) based features and k-nearest neighbors (KNN) classifier [24]. Thammasan et al. applied three commonly used algorithms to classify emotional classes: a support vector machine based on the Pearson VII kernel function (PUK) kernel, a multilayer perceptron (MLP) with one hidden layer, and C4.5 [25]. Recently, various deep learning (DL) approaches were investigated for EEG-based emotion classification. The standard deep belief networks (DBNs) were employed by Wand and Shang to extract features from raw physiological data to recognize the levels of arousal, valence, and liking [26]. In reference [27], two types of deep learning approaches, stacked denoising autoencoder and deep belief networks, were applied as feature extractors for the affective states classification problem using EEG signals. Li et al. designed a hybrid deep learning model that combines the convolutional neural network (CNN) and recurrent neural network (RNN) to extract EEG features [28].

Compared with the traditional machine learning methods, DL has achieved the promising results. However, there still exist two challenges in the multichannel EEG signals based emotion recognition. Firstly, seeing that time domain characteristics, frequency domain characteristics, and time-frequency characteristics of EEG signals contain salient information related to emotional states, naturally the complementarity between different types of features derived from these domain characteristics, respectively, is considered. Thus, feature extraction and feature fusion of multichannel EEG signals in time domain, frequency domain, and time-frequency domain need to be investigated to achieve better performance. Generally, a simple deep model such as DBN or CNN can abstract the intermediate representations of multichannel EEG features and achieve feature fusion at the feature level [29]. Nevertheless, in view of the fact of the high dimensionality and limited training samples of the physiological data, too many nodes in each layer of the deep network will lead to the model overfitting problem. Secondly, capturing the correlation information between different channels of EEG signal and extracting depth correlation feature, which are ignored by the researchers, needs to be taken into consideration when performing feature fusion using the deep model.

To address the two issues mentioned above, an integrated deep learning framework composed of DBN-GC is proposed in this paper. As a special nerve cell in human brain, glia cell can transmit signals to neurons and other glia cells. Therefore, researchers paid attention to the characteristics of glia cells and applied it to the artificial neural networks [30, 31]. In the framework, raw multidomain features are obtained from multichannel EEG signals. Then, the intermediate representations of the raw multidomain features are separately extracted by member DBN-GC, in which glia chains work for mining interchannel correlation and help to optimize learning process. Finally, a discriminative RBM is used to obtain the emotion predictions. In the experiment, the effectiveness of our method is validated on the multichannel EEG data in DEAP dataset, which is a widely used for emotion recognition.

The rest of this paper is organized as follows. A detailed description of the proposed deep learning framework based on DBN-GC is presented in Section 2. The experimental results and discussions are reported in Section 3. The last Section 4 briefly concludes the work.

## 2. Methods

### 2.1. Database

**Figure 1:**
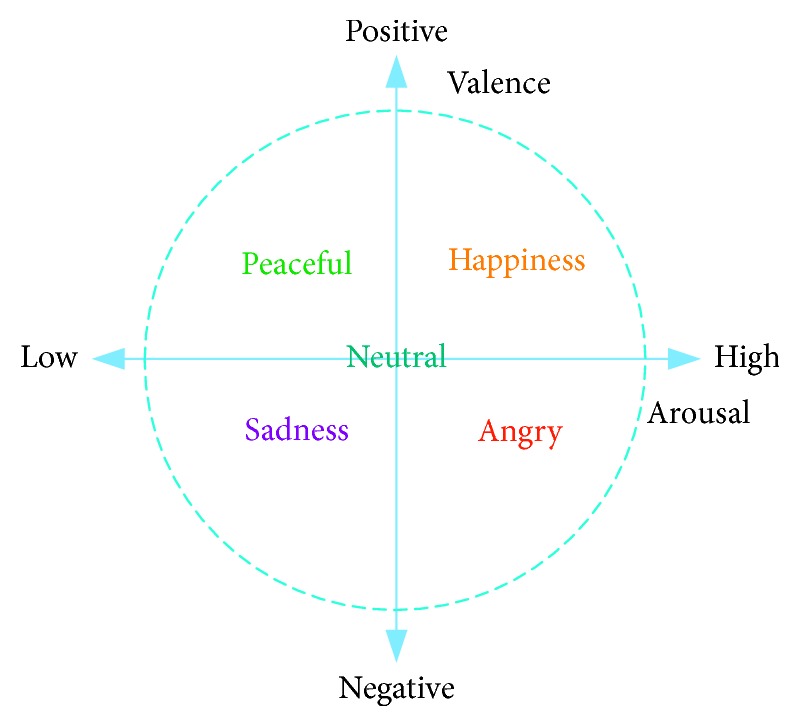
Arousal-valence plane.

In this research, the DEAP dataset is used for emotion analysis. DEAP is an open source dataset developed by the research team at Queen's University in Marie, London [32]. It mainly recorded the multimodal physiological signals produced by 32 volunteers under the stimulus of the selected videos. The multimodal physiological signals include the EEG and peripheral physiological signals. Each volunteer needed to watch 40 one-minute long videos. While each video was presented, the EEG and peripheral physiological signals of volunteers were recorded synchronously. It should be noted that the EEG was recorded from 32 sites (Fp1, AF3, F3, F7, FC5, FC1, C3, T7, CP5, CP1, P3, P7, PO3, O1, Oz, Pz, Fp2, AF4, Fz, F4, F8, FC6, FC2, Cz, C4, T8, CP6, CP2, P4, P8, PO4, and O2). Finally, the subjective-ratings of arousal, valence, liking, and dominance on a scale of 1–9 were provided. In this study, we only focus on arousal and valence scales. Thus, a two-dimensional emotional model (illustrated in Figure 1) can be built, where the two dimensions are arousal and valence, respectively. We divide and label the trials into two classes for valence and arousal, respectively (pleasant: >5, unpleasant: ≤5; aroused: >5, relaxed: ≤5).

### 2.2. Data Preprocessing and Feature Extraction

In this study, only EEG signals are employed for the emotion recognition. EEG signals recorded with 512 Hz sampling frequency are downsampled to 128 Hz. Then, filtering is implemented by a band-pass filter with cutoff frequencies of 4.0 and 45.0 Hz.

In order to make full use of the salient information regarding the emotional states in EEG signals, four types of raw features which characterizing the information in time domain, frequency domain, and time-frequency domain, respectively, are extracted from the EEG signals [32–36], and the detailed description is shown in Table 1. The 14 EEG channel pairs for achieving power differences include Fp2-Fp1, AF4-AF3, F4-F3, F8-F7, FC6-FC5, FC2-FC1, C4- C3, T8-T7, CP6-CP5, CP2-CP1, P4-P3, P8-P7, PO4-PO3, and O2-O1. Thus, the dimension of the feature vector for one instance is 664, and the label for each instance is 2-dimensional.

For one volunteer, the size of the corresponding data matrix is 40 × 664 (videos/instances × features). For all 32 volunteers, 40 × 32 = 1280 instances are available. The corresponding data matrix of each volunteer was standardized to remove the difference in feature scales.

### 2.3. Ensemble Deep Learning Framework

#### 2.3.1. Improved DBN with Glia Chains

For emotion recognition tasks, deep learning methods hypothesize that a hierarchy of intermediate representations from the EEG raw features is necessary to characterize the underlying salient information related to different emotional states. Deep belief network, which is composed of many restricted Boltzmann machines in the stacking way, has the strong ability to learn high-level representations benefiting from a deep structure-based learning mechanism with multiple hidden layers.

**Figure 2:**
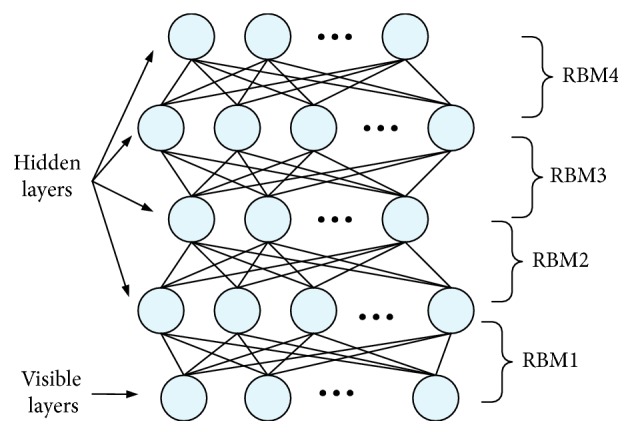
Structure of deep belief network.

As shown in Figure 2, the output of the first RBM is used as the input of the second RBM. Similarly, the third RBM is trained on the output of the second RBM. Through this way, a deep hierarchical model can be constructed that learns features from low-level features to obtain the high-level representation.

In view of the fact that there are no interconnections among the neural units of DBN in the same layer, it is hard to exploit the mutual information of different neural units in the same layer. This means that DBN is hard to work for mining interchannel and interfrequency correlation information from multichannel EEG signals in the emotion recognition tasks. Considering this, an improved DBN with glia chains is introduced in this paper.

**Figure 3:**
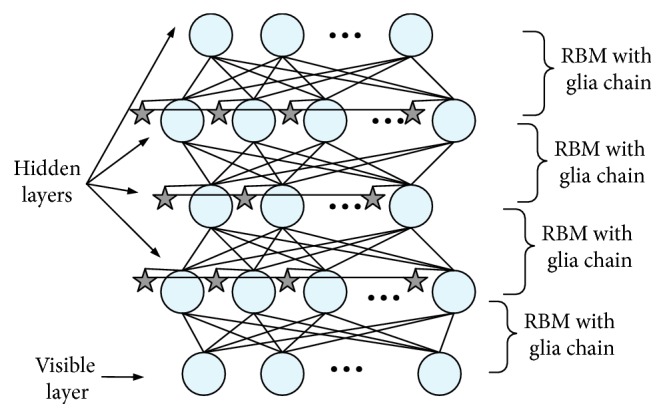
Structure of deep belief network with glia chains.

**Figure 4:**
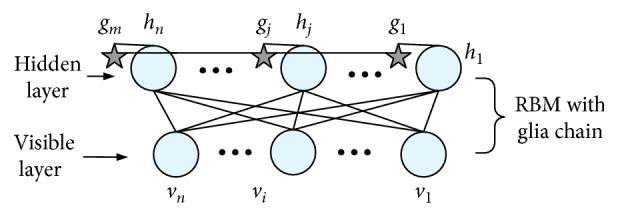
Structure of RBM with glia chain.

The structure of DBN-GC can be seen from Figure 3. In addition to the two level units of each RBM, there is a group of glia cells represented by stars and linked into a chain structure. Each glia cell is also connected to a unit in the hidden layer of RBM, as shown in Figure 4. There is no weight between the glia cells and the corresponding hidden units. The effect of all glia cells in the training process can be directly applied to the hidden units, and the outputs of the hidden layer nodes can be adjusted accordingly. Through the connection of glia cells, each glia cell can also transmit activated signal to other glia cells and adjust the glia effect of other glia cells.

For example, if the output of a hidden unit *h*_1_ is higher than the prespecified threshold, the corresponding glia cell *g*_1_ will be activated and then, a signal is transmitted to the glia cell *g*_2_. When the signal is passed to *g*_2_, the glia cell *g*_2_ will be activated, no matter whether or not the output of the hidden unit *h*_2_ reached the prespecified threshold. Then glia cell *g*_2_ will produce the second signal to spread. Meanwhile, the signal generated by *g*_1_ will continue to spread. In order to simplify the calculation, all signals produced by glia cells are propagated along the specific direction of the glia chain. That is, the signals are transmitted from the first glia cell on the chain to the last.

For RBM with a glia chain, the output rule of hidden units is updated as follows:

(1) $$\begin{array}{c} h_{j}=\sigma \left(h_{j}^{*}+\alpha \cdot g_{j}\right), \end{array}$$

where *h*_*j*_^*∗*^ is the output value of the hidden node *j* before the output rule is updated, *g*_*j*_ is the glia effect value of the corresponding glia cell, *α* is the weight coefficient of glia effect value, and *σ*() is the sigmoid function. The weight coefficient *α* is set manually, which can control the effect of glia effect on the hidden units. *h*_*j*_^*∗*^ can be calculated as

(2) $$\begin{array}{c} h_{j}^{*}=\underset{i}{\sum }W_{ij}v_{i}+c_{j}, \end{array}$$

where *W*_*ij*_ is the connection weight of the visual unit *i* and the hidden unit *j*, *v*_*i*_ is the state value of the visible unit *i*, and *c*_*j*_ is the bias value of the hidden unit *j*. Instead of random sampling, activation probability is employed as output for each hidden unit, which can reduce sampling noise and speed up learning.

The glia effect value of glia cell *g*_*j*_ is defined as

(3) $$\begin{array}{c} g_{j}\left(t\right)=\left\{\begin{array}{cc} 1, & \left(h_{j}^{*}>\theta \;\cup \;g_{j-1}\left(t-1\right)=1\right)\;\cap \;\left(t_{j}<T\right),\\ \beta g_{j}\left(t-1\right), & \text{others,} \end{array}\right. \end{array}$$

where *θ* is the prespecified threshold, *T* is an unresponsive time threshold after activation, and *β* represents the attenuation factor. Every time, the signal produced by an activated glia cell is passed to the next glia cell. The activation of a glia cell will depend on whether the output of the corresponding hidden unit reaches the prespecified threshold *θ* or whether the previous glia cell conveys a signal to it. Meanwhile, the difference *t*_*j*_ between its last activation time and the current time must be less than *T*. If the glia cell is activated, it will transmit a signal to the next glia cell; otherwise, it will not produce signals, and its glia effect will gradually decay.

After integrating the glia cell mechanism, the learning algorithm of RBM is improved and the pseudocodes of the learning algorithm are listed in Algorithm 1.

The training process of a DBN-GC, which is similar to that of DBN, consists of 2 steps: pretraining and fine-tuning. Glia cell mechanism only acts on the pretraining process. In the pretraining phase, a greedy layer-wise unsupervised method is adopted to train each RBM and the hidden layer's output of the previous RBM is used as the visible layer's input of the next RBM. In the fine-tuning phase, back propagation is performed to fine-tune the parameters of the DNB-GC.

#### 2.3.2. DBN-GC-Based Ensemble Deep Learning Model

**Figure 5:**
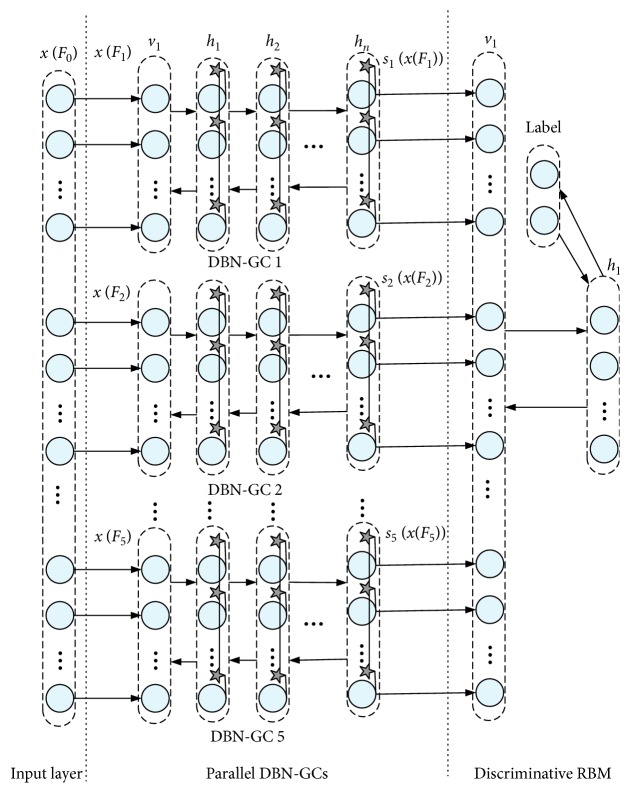
Architecture of DBN-GC-based ensemble deep learning model.

Considering that the raw EEG features in Table 1 may share different hidden properties across different time domain and frequency domain modalities, we proposed a DBN-GC-based ensemble deep learning model which implements a DBN-GC-based network on homogenous feature subset independently. The feature vectors derived from different feature subsets are fed into the corresponding DBN-GC, respectively, and the higher feature abstractions of each feature subset are obtained as the outputs of the last hidden layer in the corresponding DBN-GC. Then, a discriminative RBM is built upon the combined higher feature abstractions. The network architecture is illustrated in Figure 5. The ensemble deep learning model consists of three parts: the input layer, five parallel DBN-GCs, and a discriminative RBM.

The overall raw EEG feature set in Table 1 can be defined as *F*_0_, which is split into five nonoverlapped physiological feature subsets: *F*_1_, *F*_2_, *F*_3_, *F*_4_, and *F*_5_. The statistical measures from time domain construct subset *F*_1_, and the multichannel EEG PSDs construct subset *F*_2_. Another subset *F*_3_ is built by EEG power differences. In view of the heterogeneity of multichannel HHS features in time domain and frequency domain, the HHS features can be grouped into two subsets, *F*_4_ and *F*_5_, indicating squared amplitude and instantaneous frequency, respectively.

Define feature vectors formed by the feature subset *F*_*i*_ as *x*(*F*_*i*_). The features in *F*_0_ form the input vector *x*(*F*_0_) of the ensemble deep learning framework, which is fed into the input layer. Then, the input vector *x*(*F*_0_) is split into five subvectors: *x*(*F*_1_), *x*(*F*_2_), *x*(*F*_3_), *x*(*F*_4_), and *x*(*F*_5_). The five subvectors are the input to the corresponding DBN-GC, respectively. The five DBN-GC based deep models are built for learning the hidden feature abstractions of each raw EEG feature subset, and the hidden feature abstractions are described as *s*_1_(*x*(*F*_1_)), *s*_2_(*x*(*F*_2_)), *s*_3_(*x*(*F*_3_)), *s*_4_(*x*(*F*_4_)), and *s*_5_(*x*(*F*_5_)). *s*_*i*_(*x*(*F*_*i*_)) is the output vector of the last hidden layer of the corresponding DBN-GC *i*. Then, *s*_1_(*x*(*F*_1_)), *s*_2_(*x*(*F*_2_)), *s*_3_(*x*(*F*_3_)), *s*_4_(*x*(*F*_4_)), and *s*_5_(*x*(*F*_5_)) are merged into a vector, which is fed into the discriminative RBM to recognize emotion states.

When building the DBN-GC-based ensemble deep learning model, the five DBN-GCs are trained firstly. An additional two-neuron output layer that corresponds to binary emotions is added when training each DBN-GC. Then, the discriminative RBM is built upon the combined higher feature abstractions derived from the five DBN-GCs. To determine the DBN-GCs' hyperparameters, different combinations of hyperparameters are tested and the parameter combination with the minimal recognition error is adopted.

## 3. Results and Discussion

In view of the limited sample size in the dataset, cross-validation techniques are adopted in the experiments. The ensemble deep learning model is trained and tested via 10-fold cross-validation technique with a participant-specific style. For each of the 32 volunteers, the corresponding 40 instances are divided into 10 subsets. 9 subsets (36 instances) are assigned to the training set and the remaining 1 (4 instances) is assigned to the test set. The above process is repeated 10 times until all subsets are tested.

### 3.1. Comparison between DNB and DBN-GC

In order to study the learning performance of DBN-GC, we first use three feature subsets (*F*_6_, *F*_7_, and *F*_8_) to train DBNs and DBN-GCs, respectively. The three subsets are given as follows: *F*_6_ = *F*_1_, *F*_7_ = *F*_2_ ∪ *F*_3_, and *F*_8_ = *F*_4_ ∪ *F*_5_. The three feature subsets represent time domain characteristics, frequency domain characteristics, and time-frequency characteristics, respectively. A DBN and a DBN-GC are trained by the same feature subset, and they have the same hyperparameters, as shown in Table 2. In addition, the parameters of the DBN and the DBN-GC which share the same feature subset, such as learning rate, are all set to the same value. The six models perform the same emotion recognition task, and the metrics for recognition performance adopt accuracy and F1-score.

**Figure 6:**
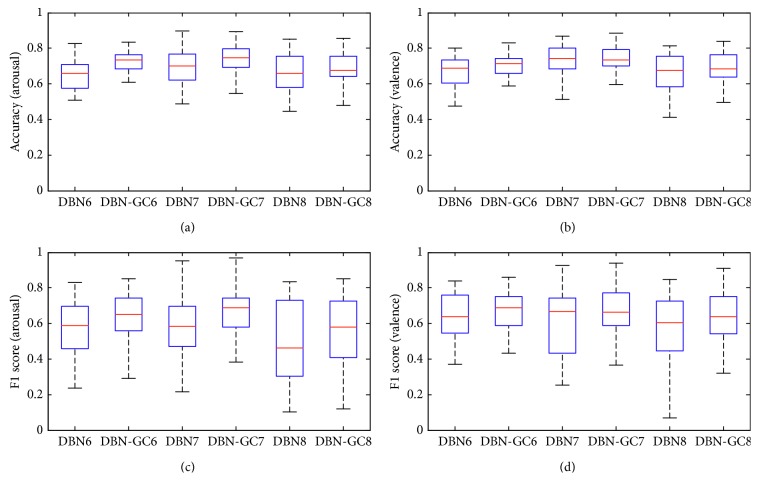
Comparison of emotion recognition results between DBN and DBN-GC.

The detailed recognition performance comparisons on arousal and valence dimensions are illustrated in Figure 6.

Each column represents the statistical results of 32 participants. Figures 6(a) and 6(c) show the classification accuracy and F1-score of arousal dimension. Figures 6(b) and 6(d) show the classification accuracy and F1-score of valence dimension. As we can see from Figure 6, no matter which feature subset is used, the DBN-GC model greatly outperforms the corresponding baseline DNN model with a higher median of accuracy and F1-score and a relatively low standard deviation. In the three DBN-GC models, DBN7 which is built by the feature subset *F*_7_ achieves the highest recognition accuracy (0.7242 on arousal and 0.7310 on valence) and F1-score (0.6631 on arousal and 0.6775 on valence). The results show that the frequency domain characteristics of EEG signals can provide salient information regarding emotion states.

The results validate that the glia chain can improve the learning performance of the deep structure. Through the glia chains, the hidden layer units in DBN-GC can transfer information to each other and the DBN-GC model can obtain the correlation information between the same hidden layer units. Thus, the improved DNB model can learn more discriminative features. For EEG-based emotion recognition task, the DNN-GC can mine interchannel correlation and utilize interchannel information, which is often ignored by other emotion recognition studies.

### 3.2. Results of the DBN-GC-Based Ensemble Deep Learning Model

Then, the proposed DBN-GC-based ensemble deep learning model is employed to perform the emotion recognition task. Each parallel DBN-GC in the ensemble deep learning model has 3 hidden layers. The numbers of hidden neuron of each parallel DBN-GC are listed in Table 3. Through the five parallel DBN-GCs, the samples' feature dimensionality is reduced from 664 to 350.

Table 4 compares the average recognition performance between the proposed DBN-GC-based ensemble deep learning model and several deep learning-based studies on the same database. Specifically, Tripathi et al. divided the 8064 readings per channel into 10 batches. For each batch, 9 characteristics, such as mean and variance were extracted. Then, a deep neural network and a convolutional neural network were used for emotion recognition [29]. Li et al. proposed the frame-based EEG features through wavelet and scalogram transform and designed a hybrid deep learning model which combined CNN and RNN [37]. In addition, Li et al. also trained a two-layer DBN to extract high-level features for each channel, and then, a SVM with RBF kernel is employed as the classifier [38]. Wang and Shang presented the DBN-based system that extracted features from raw physiological data and 3 classifiers were built to predict emotion states [26]. In view of that the above studies did not introduce F1-score as the metrics for recognition performance, the average recognition performance of the proposed model is also compared with that of reference [32]. In this reference, Koelstra et al. analyzed the central nervous system (CNS), peripheral nervous system (PNS), and multimedia content analysis features for emotion recognition. Considering the proposed DBN-GC-based method in this paper is based on the EEG signal; Table 4 only lists the recognition results of the CNS feature-based single modality in reference [32]. The DEAP dataset is used in all references in Table 4, and the trials are divided into two classes for valence and arousal, respectively (ratings divided as more than 5 and less than 5) in all references in Table 4.

As can be seen from Table 4, the performance of the DBN-GC-based ensemble deep learning model regarding the recognition accuracy outperforms most of the above deep classifiers. Meanwhile, the F1-scores achieved by the proposed model are obviously superior to 0.5830 and 0.5630 reported by reference [32]. The proposed method provides 0.7683 mean recognition accuracy (MRA) on valence, which is lower than the highest MRA reported on valence (0.8141). This may be due to that the CNN model proposed by Tripathi et al. benefits from sufficient training and validating instances. In addition, the performance of the ensemble deep learning model also outperforms the three DBN-GCs in Table 3 which are trained by a single-feature subset. This indicates that time domain characteristics, frequency domain characteristics, and time-frequency characteristics of EEG signals should be complementary in emotion recognition, and the proposed method can integrate different types of characteristics effectively.

### 3.3. Parameter Selection

**Figure 7:**
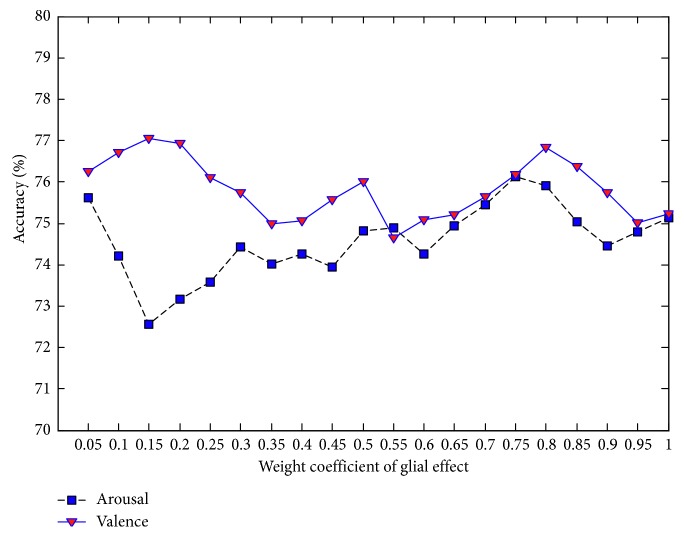
MRA of the proposed ensemble deep learning model with different values of glia effect weight.

**Figure 8:**
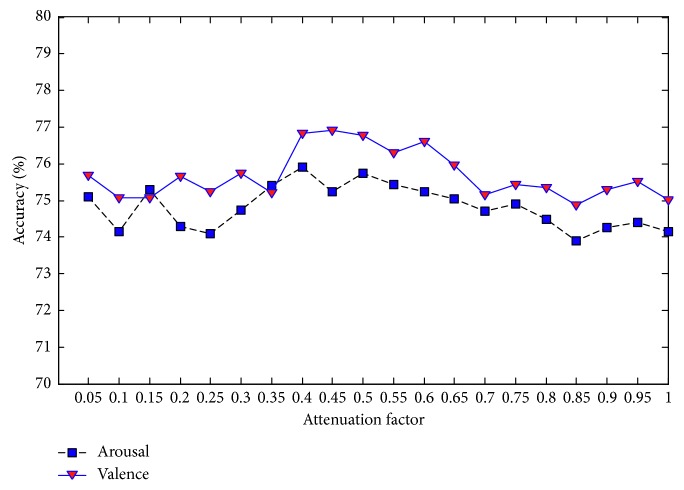
MRA of the proposed ensemble deep learning model with different values of attenuation factor.

**Figure 9:**
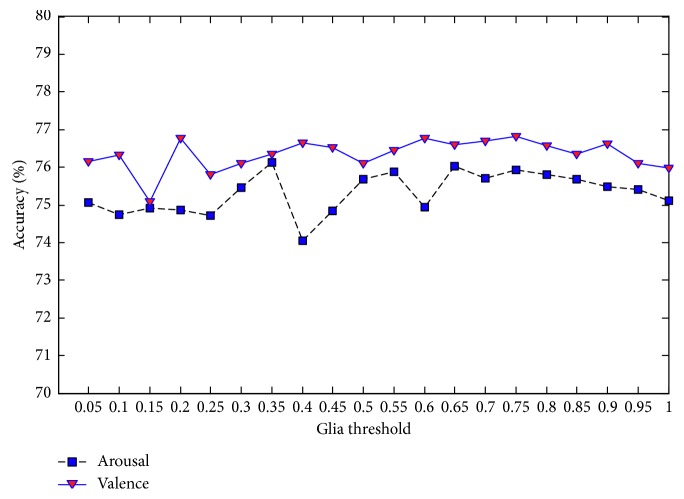
MRA of the proposed ensemble deep learning model with different values of glia threshold.

Each parallel DBN-GC in the proposed ensemble deep learning model contains three important parameters: the weight coefficient of glia effect value *α*, the attenuation factor *β*, and the glia threshold *θ*. These three parameters will determine the effect of glia cells on DBN and then affect the performance of the proposed model. Since there is no self-adaptive adjustment method, the three parameters are set manually and take 20 values in the range of 0 to 1, respectively, with the interval 0.05. Under the different values of each parameter, the MRA on valence and the MRA on arousal are taken as the final evaluation basis, and the analysis results are shown in Figures 7–9.

As can be seen from Figure 7, when the glia effect weight is between 0.05 and 1, the MRA on arousal as well as the MRA on valence fluctuates continuously. The highest MRA on arousal (76.12%) is obtained as the glia effect weight is set to 0.75. For MRA on valence, the higher values will appear when the weight value is close to 0.15 or 0.80. Taking into account these two indicators simultaneously, it is appropriate to set the weight coefficient to 0.80.

Figure 8 shows the results of arousal classification and valence classification with different values of the attenuation factor. When the value of attenuation factor is between 0.05 and 0.35, the MRA on arousal as well as the MRA on valence fluctuates greatly. With the attenuation factor increased to 0.4, both the MRA on arousal and the MRA on valence increase rapidly. Once the attenuation factor exceeds 0.5, the two MRAs have been decreasing slowly. Thus, it is appropriate to set the attenuation factor to 0.40 or 0.50.

Figure 9 shows the results of arousal classification and valence classification with different values of glia threshold. Although the highest value of MRA on arousal occurs with the glia threshold set to 0.35, the MRA is more stable when the glia threshold is between 0.65 and 1. For the MRA on valence, its value has been rising slowly when the attenuation factor exceeds 0.25. It is appropriate that the glia threshold is within the range of 0.70 to 0.80.

## 4. Conclusions

In this paper, we presented an ensemble deep learning model which integrates parallel DBN-GCs and a discriminative RBM for emotion recognition. The interchannel correlation information from multichannel EEG signals, which is often neglected, contains salient information regarding to emotion states, and the chain structure of glia cells in DBN-GC has the ability in mining interchannel correlation information. In addition, the time domain characteristics, frequency domain characteristics, and time-frequency characteristics of EEG signals should be complementary for emotion recognition, and the ensemble deep learning framework benefits from the comprehensive fusion of multidomain feature abstractions. The reliability of the DBN-GC and the ensemble deep learning framework-based fusion methods is validated by the experiments based on DEAP database.

## Figures and Tables

**Algorithm 1:**
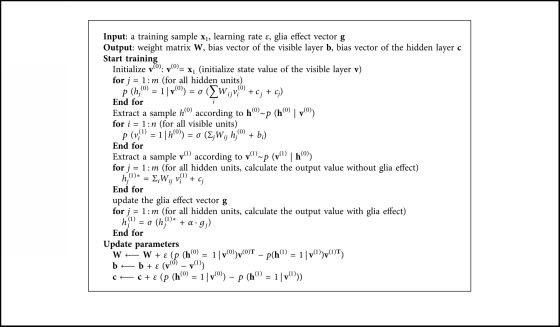
Pseudocodes for training the RBM with glia chain.

**Table 1:** Detailed description of raw EEG features.

Index	Type	Domain	Notations of the extracted features
No. 1–128	Statistical measures	Time domain	Mean, variance, zero-crossing rate, and approximate entropy of 32 EEG channels (4 features × 32 channels)
No. 129–288	Power features	Frequency domain	Average PSD in theta (4–8 Hz), slow-alpha (8–10 Hz), alpha (8–12 Hz), beta (12–30 Hz), and gamma (30–45 Hz) bands for all EEG channels (5 power × 32 channels)
No. 289–344	Power differences	Frequency domain	Difference of average PSD in theta, alpha, beta, and gamma bands for 14 EEG channel pairs between right and left scalp (4 power differences × 14 channel pairs)
No. 345–664	HHS features	Time-frequency domain	Average values of squared amplitude and instantaneous frequency of HHS-based time–frequency representation in delta (1–4 Hz), theta (4–8 Hz), alpha (8–12 Hz), beta (12–30 Hz), and gamma (30–45 Hz) bands for all EEG channels (2 features × 5 bands × 32 channels)

**Table 2:** Hyperparameters of DBNs and DBN-GCs.

	*F*_6_		*F*_7_		*F*_8_	
	DBN6	DBN-GC6	DBN7	DBN-GC7	DBN8	DBN-GC8
*v*_1_	128	128	216	216	320	320
*h*_1_	100	100	200	200	300	300
*h*_2_	80	80	135	135	280	280
*h*_3_	45	45	90	90	125	125
*o*_1_	2	2	2	2	2	2

Note: *v*_1_ represents the input layer, *o*_1_ represents the output layer, and *h*_*i*_ represents the *i*-th hidden layer.

**Table 3:** Hyperparameters of each parallel DBN-GC in the ensemble deep learning model.

	*h*_1_	*h*_2_	*h*_3_
DBN-GC1	110	105	90
DBN-GC2	145	145	120
DBN-GC3	55	55	45
DBN-GC4	120	55	45
DBN-GC5	155	80	50
Sum	585	440	350

**Table 4:** Average recognition performance comparison between the DBN-GC-based ensemble deep learning model and several reported studies.

	Arousal		Valence	
	Accuracy	F1-score	Accuracy	F1-score
Deep neural network [29]	0.7313	—	0.7578	—
Convolutional neural network [29]	0.7336	—	0.8141	—
CNN/RNN [37]	0.7412	—	0.7206	—
DBN-SVM [37]	0.6420	—	0.5840	
Wang and Shang [26]	0.5120	—	0.6090	—
CNS feature-based single modality [32]	0.6200	0.5830	0.5760	0.5630
DBN-GC-based ensemble deep learning model	0.7592	0.6931	0.7683	0.7015

## Data Availability

The DEAP dataset used in our manuscript is a dataset for emotion analysis using electroencephalogram (EEG) and physiological and video signals. The DEAP dataset is available at http://www.eecs.qmul.ac.uk/mmv/datasets/deap/. Anyone interested in using this dataset will have to print, sign, and scan an EULA (end-user license agreement) and return it via e-mail. Then, a username and password to download the data will be provided. The dataset was first presented in reference [32]. DEAP: a database for emotion analysis using physiological signals.

## References

[1] Pedro M.-F., Arquimedes M.-P., Antoni J.-C., Rubio J. M. B. (2014). Evaluating the research in automatic emotion recognition. *IETE Technical Review*.

[2] Chen M., He X., Yang J., Zhang H. (2018). 3-D convolutional recurrent neural networks with attention model for speech emotion recognition. *IEEE Signal Processing Letters*.

[3] Liu Z.-T., Xie Q., Wu M., Cao W.-H., Mei Y., Mao J.-W. (2018). Speech emotion recognition based on an improved brain emotion learning model. *Neurocomputing*.

[4] Anaqnostopoulos C.-N., Iliou T., Giannoukos I. (2015). Features and classifiers for emotion recognition from speech: a survey from 2000 to 2011. *Artificial Intelligence Review*.

[5] Kahou S.-E., Michalski V., Konda K. Recurrent neural networks for emotion recognition in video.

[6] Huang Y., Yang J., Liao P., Pan J. (2017). Fusion of facial expressions and EEG for multimodal emotion recognition. *Computational Intelligence and Neuroscience*.

[7] Wang X.-W., Nie D., Lu B.-L. (2014). Emotional state classification from EEG data using machine learning approach. *Neurocomputing*.

[8] Yoon H. J., Chung S. Y. (2013). EEG-based emotion estimation using Bayesian weighted-log-posterior function and perceptron convergence algorithm. *Computers in Biology and Medicine*.

[9] Abeer A.-N., Manar H., Yousef A.-O., Areej A.-W. (2017). Review and classification of emotion recognition based on EEG brain-computer interface system research: a systematic review. *Applied Sciences-Basel*.

[10] Liu Y., Sourina O. (2013). Real-time fractal-based valence level recognition from EEG. *Transactions on Computational Science XVIII*.

[11] Murugappan M., Ramachandran N., Sazali Y. (2010). Classification of human emotion from EEG using discrete wavelet transform. *Journal of Biomedical Science and Engineering*.

[12] Chai T.-Y., San W.-S., Tan C.-S., Rizon M. (2009). Classification of human emotions from EEG signals using statistical features and neural network. *International Journal of Integrated Engineering*.

[13] Frantzidis C., Bratsas C., Papadelis C., Konstantinidis E., Pappas C., Bamidis P. D. (2010). Toward emotion aware computing: an integrated approach using multichannel neurophysiological recordings and affective visual stimuli. *IEEE Transactions on Information Technology in Biomedicine*.

[14] Hjorth B. (1970). EEG analysis based on time domain properties. *Electroencephalography and Clinical Neurophysiology*.

[15] Ansari-asl K., Chanel G., Pun T. A channel selection method for EEG classification in emotion assessment based on synchronization likelihood.

[16] Kroupi E., Yazdani A., Ebrahimi T. EEG correlates of different emotional states elicited during watching music videos.

[17] Petrantonakis P. C., Hadjileontiadis L. J. (2010). Emotion recognition from EEG using higher order crossings. *IEEE Transactions on Information Technology in Biomedicine*.

[18] Petrantonakis P. C., Hadjileontiadis L. J. (2010). Emotion recognition from brain signals using hybrid adaptive filtering and higher order crossings analysis. *IEEE Transactions on Affective Computing*.

[19] Verma G. K., Tiwary U. S. (2014). Multimodal fusion framework: a multiresolution approach for emotion classification and recognition from physiological signals. *NeuroImage*.

[20] Hadjidimitriou S. K., Hadjileontiadis L. J. (2012). Toward an EEG-based recognition of music liking using time-frequency analysis. *IEEE Transactions on Biomedical Engineering*.

[21] Akin M. (2002). Comparison of wavelet transform and FFT methods in the analysis of EEG signals. *Journal of Medical Systems*.

[22] Murugappan M., Rizon M., Yaacob S. (2007). EEG feature extraction for classifying emotions using FCM and FKM. *International Journal of Computers and Communications*.

[23] Samara A., Menezes M.-L.-R., Galway L. Feature extraction for emotion recognition and modeling using neurophysiological data.

[24] Jadhav N., Manthalkar R., Joshi Y. Electroencephalography-Based emotion recognition using gray-level co-occurrence matrix features.

[25] Thammasan N., Moriyama K., Fukui K.-i., Numao M. (2016). Familiarity effects in EEG-based emotion recognition. *Brain Informatics*.

[26] Wang D., Shang Y. (2013). Modeling physiological data with deep belief networks. *International Journal of Information and Education Technology*.

[27] Xu H., Plataniotis K.-N. Affective states classification using EEG and semi-supervised deep learning approaches.

[28] Li X., Song D., Zhang P., Hou Y., Hu B. (2017). Deep fusion of multi-channel neurophysiological signal for emotion recognition and monitoring. *International Journal of Data Mining and Bioinformatics*.

[29] Tripathi S., Acharya S., Sharma R.-D. Using deep and convolutional neural networks for accurate emotion classification on DEAP dataset.

[30] Ikuta C., Uwate Y., Nishio Y. Multi-layer perceptron with positive and negative pulse glia chain for solving two-spirals problem.

[31] Geng Z.-Q., Zhang Y.-K. (2016). An improved deep belief network inspired by glia chains. *Acta Automatica Sinica*.

[32] Koelstra S., Muehl C., Soleymani M. (2012). DEAP: a database for emotion analysis using physiological signals. *IEEE Transaction on Affective Computing*.

[33] Khezri M., Firoozabadi M., Sharafat A. R. (2015). Reliable emotion recognition system based on dynamic adaptive fusion of forehead biopotentials and physiological signals. *Computer Methods and Programs in Biomedicine*.

[34] Atkinson J., Campos D. (2016). Improving BCI-based emotion recognition by combining EEG feature selection and kernel classifiers. *Expert Systems with Applications*.

[35] Li C., Xu C., Feng Z. (2016). Analysis of physiological for emotion recognition with the IRS model. *Neurocomputing*.

[36] Yin Z., Zhao M.-Y., Wang Y.-X., Yang J., Zhang J. (2017). Recognition of emotions using multimodal physiological signals and an ensemble deep learning model. *Computer Methods and Programs in Biomedicine*.

[37] Li X., Song D.-W., Zhang P. Emotion recognition from multi-channel EEG data through convolutional recurrent neural network.

[38] Li X., Zhang P., Song D.-W. EEG based emotion identification using unsupervised deep feature learning.

